# Facile Synthesis of Rhodium‐Based Nanocrystals in a Metastable Phase and Evaluation of Their Thermal and Catalytic Properties

**DOI:** 10.1002/smtd.202401143

**Published:** 2024-10-22

**Authors:** Quynh N. Nguyen, Kei Kwan Li, Yong Ding, Annemieke Janssen, Zhennan Huang, Miaofang Chi, Younan Xia

**Affiliations:** ^1^ School of Chemistry and Biochemistry Georgia Institute of Technology Atlanta GA 30332 USA; ^2^ School of Materials Science and Engineering Georgia Institute of Technology Atlanta GA 30332 USA; ^3^ Center for Nanophase Materials Science Oak Ridge National Laboratory Oak Ridge TN 37831 USA; ^4^ The Wallace H. Coulter Department of Biomedical Engineering Georgia Institute of Technology and Emory University Atlanta GA 30332 USA

**Keywords:** crystal phase, nanocrystals, polymorphism, rhodium, seed‐mediated growth

## Abstract

Controlling the polymorphism of metal nanocrystals is a promising strategy for enhancing properties and discovering new phenomena. However, previous studies on Rh nanocrystals have focused on their thermodynamically stable face‐centered‐cubic (fcc) phase. Herein, a facile synthesis of Rh‐based nanocrystals featuring the metastable hexagonal close‐packed (hcp) phase is reported by using Ru seeds in their native hcp phase to template the deposition of Rh atoms. The success of such phase‐controlled synthesis relies on the templating effect promoted by the small lattice mismatch between Ru and Rh and the slow dropwise titration of the precursor at an elevated temperature, ensuring the layer‐by‐layer growth mode and thus the formation of a conformal hcp‐Rh shell. Faster injection rate of Rh(III) precursor leads to the formation of a rough Rh shell in the conventional fcc phase due to accelerated reaction kinetics. Considering both thermodynamic and kinetic aspects of this system, the hcp‐Rh phase is favored when the low surface energy from smooth overlayers balances the high bulk energy of the metastable phase, achieved through tight control of reaction rates and deposition patterns. These Ru_hcp_@Rh_hcp_ core–shell nanocrystals demonstrate thermal stability up to 400 °C, while exhibiting higher catalytic activity toward ethanol oxidation reaction compared to Ru_hcp_@Rh_fcc_ counterparts.

## Graphical abstract

Rh‐based nanocrystals featuring the metastable hexagonal close‐packed (hcp) phase are synthesized using hcp‐Ru seeds. Slow dropwise titration of the precursor at elevated temperatures promotes a layer‐by‐layer growth, forming a conformal hcp‐Rh shell with low surface energy that balances the high bulk energy. These Ru@Rh core–shell nanocrystals with an hcp phase exhibit thermal stability up to 400 °C and enhanced catalytic activity toward ethanol oxidation.

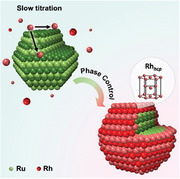

## 1 Introduction

The performance of noble‐metal nanocrystals in a wide array of applications has been traditionally optimized by fine‐tuning physical attributes such as size, shape, and defects to influence the surface atomic arrangements.^[^ ^1^, ^2^, ^3^ ^]^ Recent research endeavors have pivoted towards manipulating the atomic arrangement within the bulk structure, particularly the crystal phase, of these nanocrystals to augment their mechanical, electrical, magnetic, optical, and catalytic properties.^[^ ^4^, ^5^, ^6^, ^7^ ^]^ Precise control over polymorphism—the ability of nanocrystals to crystallize in various phases—has emerged as a promising strategy to boost catalytic efficiency by modifying how the reaction intermediates interact with surface atoms.^[^ ^8^, ^9^, ^10^ ^]^ A notable example is the superior performance of metastable face‐centered‐cubic (fcc)‐Ru octahedral nanoframes over their hexagonal close‐packed (hcp) counterparts in reactions such as the dehydrogenation of ammonia borane and reduction of 4‐nitrophenol.^[^ ^11^, ^12^ ^]^ Similarly, the Ru_hcp_@Pd_hcp_ nanocrystals outperformed Ru_hcp_@Pd_fcc_ in formic acid oxidation, demonstrating higher specific activity and peak potential due to the weaker CO_2_ binding and minimized surface poisoning.^[^ ^13^ ^]^ These findings manifest the potential of phase‐controlled metal nanocrystals as catalysts for structure‐sensitive reactions.

Metastable phases have been achieved for a number of noble metals, including hcp with a stacking sequence of ABAB for Au, Pd, and Pt;^[^ ^13^, ^14^, ^15^, ^16^ ^]^ 4H with a stacking sequence of ABCB for Au and Ag;^[^ ^7^, ^17^ ^]^ and fcc with a stacking sequence of ABCABC for Ru.^[^ ^18^ ^]^ Additionally, heterophased structures combing fcc and hcp have been realized for Ag and Ru.^[^ ^19^, ^20^ ^]^ Despite this progress, research on Rh nanocrystals has predominantly concentrated on their thermodynamically stable fcc phase, as dictated by their inherent bulk characteristics. Processing Rh as colloidal nanocrystals with uniform size while featuring a high percentage of metastable phases remains in its infancy. Early research efforts introduced two distinct methods for fabricating hcp‐Rh nanocrystals, each presenting unique challenges regarding scalability and phase purity.^[^ ^21^ ^]^ The first method leveraged electron beam‐induced decomposition of Rh monolayers, leading to the aggregation of some atoms into hcp‐Rh nanocrystals amidst voids within the residual monolayers.^[^ ^21^ ^]^ However, this approach primarily produced a minor fraction of the targeted hcp phase, rendering it more of a theoretical exploration than a viable solution for large‐scale production. The second method involved the direct solvothermal reduction of a Rh(III) precursor, with borane tert‐butylamine and oleylamine serving as the reductant and solvent, respectively.^[^ ^21^ ^]^ This method yielded hcp‐Rh nanocrystals with very small sizes (less than 5 nm) and poorly defined structures, compromising their effectiveness for catalytic applications. This challenge primarily stems from the high cohesive and surface free energies of Rh, which complicate their fabrication via conventional one‐pot synthesis.

In contrast, seed‐mediated growth offers a more straightforward and versatile approach for fabricating metal nanocrystals with metastable phases.^[^ ^22^ ^]^ Decoupling the growth stage from the more intricate nucleation process, this technique leverages preformed nanocrystals that display the desired phase to guide the newly formed atoms in replicating the underlying packing sequence. The presence of a preformed seed not only results in a lower nucleation barrier but also facilitates the formation of core–shell nanocrystals where the deposited overlayers inherit the crystal phase of the seeds.^[^ ^22^, ^23^ ^]^ This method has enabled the production of Au–Rh hierarchical hybrid nanowires featuring 4H/fcc‐Rh heterophase.^[^ ^24^ ^]^ Specifically, the vertical growth of Rh nanorods was initiated on the 4H domains and fcc twin boundaries of the Au nanowires due to the high surface energies of these sites. Such phase‐controlled epitaxial growth has also constructed an array of Rh‐based hierarchical nanostructures with a mix of metastable and native phases, such as hcp/fcc‐Rh nanorods and nanoplates on hcp/fcc‐Au nanosheets and hcp‐Pd nanocrystals.^[^ ^25^, ^26^ ^]^ Despite the success in these heterophased systems, extending this seed‐mediated method to produce Rh nanocrystals in a pure metastable hcp phase has yet to be fully explored. Given the critical role of Rh in numerous catalytic processes, such as the hydrogen evolution reaction, hydrogenation, CO oxidation, and hydrocarbonylation,^[^ ^27^, ^28^, ^29^ ^]^ developing a robust protocol for the phase‐controlled synthesis of Rh nanocrystals could unlock new possibilities in the field of catalysis.

Motivated by these opportunities and challenges, we demonstrated herein the feasibility of fabricating Rh‐based nanocrystals in the metastable hcp phase via phase‐controlled epitaxial growth on hcp‐Ru seeds. This approach was augmented by a drop‐wise titration technique to achieve uniform deposition and fine control over the thickness of the shell. The inherent hcp phase of Ru, along with the small lattice mismatch between Ru and Rh (0.7%) and the robust protocol for fabricating Ru_hcp_ nanocrystals, rendered Ru a promising template for generating Rh overlayers in a metastable hcp phase. The success of such a phase control using seed‐mediated growth relied on the slow injection rate of the precursor coupled with an elevated temperature to ensure the layer‐by‐layer deposition and thus the formation of a conformal hcp‐Rh shell. Integrating both thermodynamic and kinetic controls, the metastable hcp‐Rh was favored as the increase in bulk energy associated with the metastable phase could be offset by the reduced surface energy from smooth overlayers, a condition achieved through tight controls over the reaction rates and deposition patterns. When subjected to thermal stress, these metastable hcp‐Rh structures exhibited remarkable stability, maintaining their phase integrity up to 400 °C. As a proof‐of‐concept application, we investigated the phase–catalytic relationship of the resultant Ru@Rh nanocrystals featuring polymorphism towards ethanol oxidation reaction (EOR). Notably, Ru_hcp_@Rh_hcp_ core–shell nanocrystals showed higher activity when benchmarked against Ru_hcp_@Rh_fcc_ counterparts, highlighting the importance of polymorphism in augmenting catalytic efficiency.

## 2 Results and Discussion

### 2.1 Synthesis and Characterization of Ru@Rh Nanocrystals Featuring Polymorphism

The synthesis began with the preparation of Ru nanocrystals in the conventional hcp phase (denoted Ru_hcp_ seeds thereafter). The intrinsic hcp phase of bulk Ru, coupled with the small lattice mismatch between Ru and Rh (0.7%, 2.71 vs 2.69Å), and the robust protocol for producing Ru_hcp_ nanocrystals with controlled sizes, establishes Ru as a suitable template for facilitating the generation of Rh shell in a metastable hcp phase. The effectiveness of this phase‐controlled synthesis also requires the use of Ru_hcp_ seeds that possess thermal stability and an optimal size to sustain the surface diffusion of Rh adatoms. Therefore, we chose Ru_hcp_ seeds of ca. 6–7 nm in size as they provide sufficient surface area for Rh nucleation and conformal growth while preserving the structural integrity and phase purity crucial for further characterization and catalytic applications. Figure S1A (Supporting Information) shows a typical TEM image of the Ru nanocrystals prepared through a two‐step growth method using a modified published protocol.^[^ ^13^, ^18^ ^]^ These nanocrystals exhibited a pseudo‐spherical shape alongside a relatively uniform size distribution, with an average diameter of 6.7 ± 0.8 nm. The HRTEM image of an individual Ru_hcp_ seed taken from the [21̅10] zone axis shows the lattice fringe spacings that align well with the expected interplanar spacings for the hcp‐Ru plane (Figure S1B, Supporting Information). The XRD pattern also confirmed the hcp phase of the Ru nanocrystals, revealing three broad peaks at 2*θ* of 38.4°, 42.1°, and 44.0°, corresponding to (101̅0), (0002), and (101̅1) diffractions of hcp‐Ru (Figure S1C, Supporting Information).^[^ ^13^, ^18^ ^]^ The Ru_hcp_ seeds were used directly without further purification due to the similar reagents employed throughout the synthetic processes.

**Figure 1:**
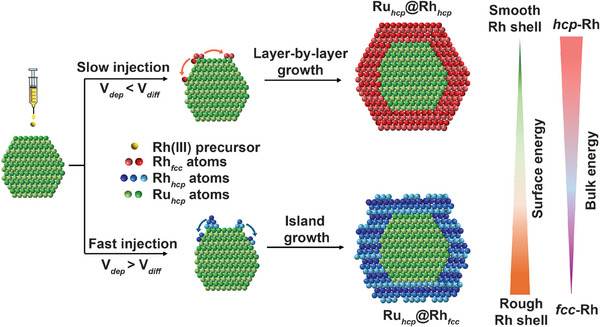
Schematic illustration showing the formation of Ru@Rh core–shell nanocrystals with different crystal phases when varying the injection rates of the precursor while keeping all other synthetic parameters the same.

Next, we conducted seed‐mediated growth to epitaxially deposit Rh shells on the Ru_hcp_ seeds for the generation of Ru@Rh nanocrystals. While conceptually straightforward, simply depositing Rh atoms on the seeds may not adequately activate the templating effect necessary for creating a perfect hcp‐Rh shell. In principle, the crystal phase of a metal nanocrystal depends on the interplay between bulk and surface energies, where a metastable phase is favored when a gain in bulk energy is balanced by a reduction in surface energy.^[^ ^12^, ^30^, ^31^ ^]^ In core–shell nanocrystals, this energy equilibrium is facilitated through a uniform overlay coating, which is in turn dictated by the pattern of atom deposition or the growth mode.^[^ ^32^ ^]^ Specifically, a layer‐by‐layer growth mode is crucial for constructing a conformal and smooth shell with low surface energy, enabling the stabilization of a metastable phase despite its higher bulk energy.^[^ ^11^, ^33^ ^]^ In contrast, the island growth mode gives rise to a rough shell with jagged edges, characterized by elevated surface energy due to the increased number of undercoordinated atoms.^[^ ^30^, ^31^ ^]^ Such an overlayer tends to revert to its native, thermodynamically stable phase with a lower bulk energy as compensation to minimize the total energy of the system. By modulating the growth mode (resulting in smooth or rough Rh shells), we varied the relative contributions from the surface and bulk energies, ultimately maneuvering the phase of Rh shells epitaxially grown on the Ru_hcp_ seeds (**Figure** **1**).

**Figure 2:**
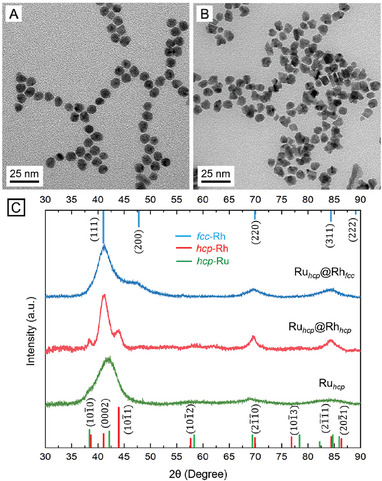
A,B) TEM images of the Ru@Rh core–shell nanocrystals synthesized with different injection rates for the Rh(III) precursor: A) 0.5 mL h^−1^ and B) 4 mL h^−1^. C) XRD patterns of Ru_hcp_@Rh_hcp_ and Ru_hcp_@Rh_fcc_ nanocrystals, together with that of the Ru_hcp_ seeds for comparison. The red, blue, and green lines correspond to the characteristic peaks of hcp‐Rh (simulated), fcc‐Rh (JCPDS No. 05‐0685), and hcp‐Ru (JCPDS No. 06‐0663), respectively.

The relationship between the rate of deposition (*V*_dep_) and the rate of surface diffusion (*V*_diff_) serves as a simple metric for controlling the growth mode of metal nanocrystals.^[^ ^34^ ^]^ To promote layer‐by‐layer growth, one can decelerate the deposition rate of atoms by slowly introducing the precursor solution into the reaction while maintaining a high temperature to promote surface diffusion of the adatoms (i.e., *V*_dep_ < *V*_diff_).^[^ ^35^ ^]^ In contrast, the atom deposition rate must exceed the surface diffusion rate (i.e., *V*_dep_ > *V*_diff_) to initiate the island growth mode and thus the formation of a rough coating on the seeds.^[^ ^36^ ^]^ Consequently, we adjusted the injection rates of the Rh(III) precursor during the synthesis of Ru@Rh core–shell nanocrystals to examine the impact of growth mode on the crystal phase. In a proof‐of‐concept experiment, an EG solution of Rh(acac)_3_ precursor was titrated at a controlled rate into a suspension of the Ru_hcp_ seeds in EG at 160 °C using a syringe pump. **Figure** **2**A,B shows typical TEM images of the nanocrystals obtained when the injection rate was set to 0.5 and 4 mL h^−1^, respectively (Figure 2 and Figure S2, Supporting Information). The as‐obtained nanocrystals had average diameters of 8.4 ± 0.7 and 8.6 ± 1.3 nm, respectively. The increase in size compared with the starting Ru_hcp_ seeds confirmed the successful deposition of Rh overlayers, which corresponded to a shell thickness of ca. 0.9 nm (equivalent to 3–4 atomic layers of Rh). Despite similar size ranges, the morphologies of the two samples exhibited clear differences, indicative of two distinct growth modes. When the injection rate was controlled at a relatively slow rate of 0.5 mL h^−1^, a limited supply of Rh atoms was generated in each droplet and deposited onto the seeds, followed by their diffusion across the entire surface at a relatively high temperature of 160 °C to form a uniform overlayer. After titrating 10 mL of the Rh(III) precursor into the reaction solution, the Ru@Rh products had a uniform size and smooth shell while retaining the quasi‐spherical morphology of most Ru_hcp_ seeds, validating the layer‐by‐layer growth mode (Figure 2A). Besides tunning the ratio of atom deposition to diffusion rate, the slow injection rate of Rh(III) precursor led to decelerated reduction kinetics, ensuring that the concentration of the newly formed Rh atoms remained below supersaturation to prevent homogeneous nucleation detrimental to phase control.^[^ ^36^, ^37^ ^]^ This effect is evident by the absence of a second population of tiny Rh particles that often adopted the native fcc phase in the as‐obtained products. Increasing the injection rate to 4 mL h^−1^ led to the formation of nanocrystals with a rough and protruding surface covered by a high density of small bumps (Figure 2B). This irregular morphology suggested a growth mode where an excess of Rh atoms accumulated on the high‐energy sites of the seeds, forming islands due to insufficient time for diffusion before the subsequent batch of atoms was introduced.

To elucidate the difference in phase as a function of injection rates and growth modes, we analyzed the XRD patterns of the resulting Ru@Rh nanocrystals (Figure 2C). Notably, the sample produced under a slow injection rate of 0.5 mL h^−1^ displayed an XRD pattern markedly different from those previously reported for monometallic fcc‐Rh nanocrystals^[^ ^36^, ^38^ ^]^ or database entries (JCPDS No. 05‐0685). However, the XRD pattern resembled that of Ru_hcp_ seeds, albeit with a subtle shift in peak position and sharpened peaks due to the compositional change and increased crystalline size. Our previous study has confirmed that the crystal structure of Ru_hcp_ seeds remained unchanged under similar reaction conditions for 22 h, ruling out any influence of Ru core crystallinity on the XRD pattern.^[^ ^37^ ^]^ A simulated diffraction pattern was generated for monometallic hcp‐Rh using lattice parameters (*a* = 2.689 Å, *c* = 4.391 Å), derived from fcc‐Rh (*a* = 3.803 Å) by employing the relationship *a*_fcc_ ≈ $\sqrt{2}$ *a*_hcp_ and *c*_hcp_ = 2$\sqrt{6}$/3 *a*_hcp_ (Figure S2, Supporting Information).^[^ ^39^ ^]^ The peak positions in the simulated diffraction pattern were calculated based on these lattice parameters and used for phase identification, without accounting for peak widths, due to the inhomogeneity in crystallite size distribution among the resulting nanocrystals. The simulated and experimental patterns exhibited good agreement, with the three characteristic peaks positioned at 38.5°, 41.1°, and 44.0° corresponding to the diffractions from (101̅0), (0002), and (101̅1) planes of hcp‐Rh, respectively (Figure 2C). The slight shift of the highest‐intensity peak to 41.4°, between the reference peaks of hcp‐Ru(0002) at 42.2° and hcp‐Rh(0002) at 41.1°, could be attributed to the interaction and averaging signals between the core and shell.^[^ ^40^ ^]^ It should be noted that the observed deviations in peak intensity from the simulated XRD pattern could be due to the texture effect, which arises when certain facets of the nanocrystals align preferentially with the substrate during the drying process, thereby affecting the relative peak intensities in the XRD pattern.^[^ ^40^, ^41^ ^]^ These results confirmed that the Rh shells inherited the hcp phase of the Ru seeds under a slow injection rate of precursor, leading to the generation of Ru_hcp_@Rh_hcp_ core–shell nanocrystals. In contrast, the XRD pattern of the sample produced at a fast injection rate of 4 mL h^−1^ displayed a distinct set of peaks characteristic of the fcc phase (Figure 2C). The emergence of two primary peaks at 41.1° and 47.5° could be assigned to (111) and (200) planes of fcc‐Rh, indicating the deposition of Rh in its native fcc phase. The residual hcp peaks from the Ru core could still be observed at 38.4° and 44.0° but notably diminished due to its low content. Overall, the XRD patterns revealed that the crystal phase of the deposited Rh depends on the growth conditions, transitioning from metastable hcp‐Rh to native fcc‐Rh phase as the injection rate of the precursor increased, thereby shifting between layer‐by‐layer and island growth modes.

**Figure 3:**
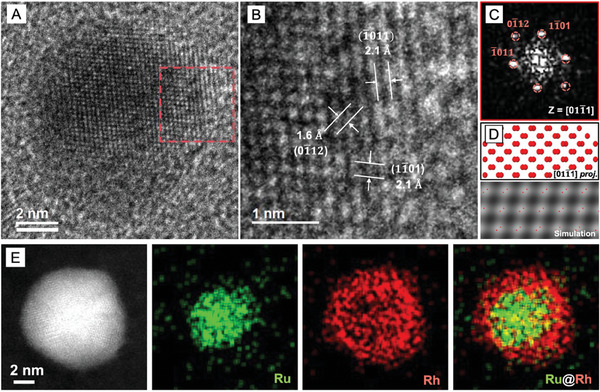
Characterizations of Ru_hcp_@Rh_hcp_ core–shell nanocrystals: A) HRTEM image, B) atomic resolution HRTEM image, and C) the corresponding FFT pattern of the red boxed region in (A), D) the projected model of Rh atoms in the hcp structure (top) and simulated HRTEM image (bottom) viewed along the $\left[01\bar{1}1\right]$ direction, and E) HAADF‐STEM image and the corresponding EDX elemental mappings of a core‐shell nanocrystal.

To support the XRD data, we employed HRTEM to confirm the crystal phases of the Ru_hcp_@Rh_hcp_ and Ru_hcp_@Rh_fcc_ nanocrystals obtained by solely varying the injection rate of the precursor. **Figure** **3**A,B shows the HRTEM image of an individual Ru_hcp_@Rh_hcp_ nanocrystal and the corresponding magnified image acquired from the surface region (red boxed). The lattice fringe spacings of 2.1 and 1.6 Å correspond to the ($\bar{1}011$) and (0$\bar{1}12$) planes of hcp‐Rh, respectively. The FFT analysis of the surface region, marked by a red box, yields a diffraction pattern that matches the hcp lattice viewed along the $\left[01\bar{1}1\right]$ direction (Figure 3C). The simulated HRTEM image with a superimposed projection of atoms presents an atomic packing pattern similar to the experimental image taken from the $\left[01\bar{1}1\right]$ zone axis, providing additional evidence supporting the proposed hcp‐Rh crystal phase (Figure 3D). The HAADF‐STEM image and the corresponding magnified image of the outer region further confirm the formation of the hcp structure by clearly showing the distinctive “ABABAB” atomic stacking sequence along the [21̅10] zone axis (Figure S4, Supporting Information). The elemental distribution of Ru and Rh was characterized using EDX mapping and line scanning of the cross‐section of the bimetallic nanocrystals (Figure 3E and Figure S5A, Supporting Information). Specifically, Ru atoms (green region) were mainly confined to the core while Rh atoms (red region) were distributed in the outermost layer, suggesting that the products are Ru_hcp_@Rh_hcp_ core–shell nanocrystals. The X‐ray photoelectron spectroscopy (XPS) spectra further validated the elemental states of Ru and Rh in the products (Figure S5B–D, Supporting Information). The characteristic singlet peak of 3p orbitals and doublet peaks of 3d orbitals of Ru, as well as 3d orbitals of Rh, were clearly resolved, with the peak of each element dominated by the zero‐valence state. These structural characterizations confirmed that conformal and smooth Rh overlayers were epitaxially grown on Ru_hcp_ seeds, which in turn adopted the hcp phase of the underlying packing sequence.

**Figure 4:**
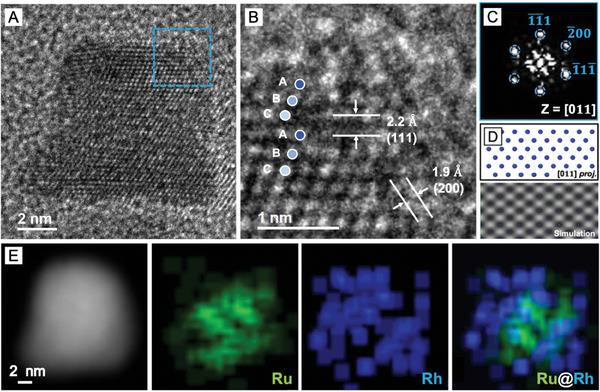
Characterizations of Ru_hcp_@Rh_fcc_ core–shell nanocrystals: A) HRTEM image, B) atomic resolution HRTEM image, and C) the corresponding FFT pattern of the blue boxed region in (A). D) The projected model of Rh atoms in the fcc structure (top) and simulated HRTEM image (bottom) viewed along the [011] direction, and E) HAADF‐STEM image and the corresponding EDX elemental mappings of a core‐shell nanocrystal.

On the other hand, the HRTEM image of an individual Ru_hcp_@Rh_fcc_ nanocrystal outlined a rough surface covered by small bumps at the corners (**Figure** **4**A). A closer inspection of the atomic‐resolution HRTEM image taken from the shell region (blue boxed) revealed the distinctive “ABCABC” atomic stacking sequence of the fcc structure along the close‐packing [011] direction (Figure 4B). The lattice fringe spacings of 2.2 and 1.9 Å align well with the interplanar spacings expected for fcc‐Rh (111) and (200) planes, respectively. The FFT pattern acquired from the shell region (blue boxed) also exhibits diffraction spots corresponding to the fcc phase viewed along the [011] direction (Figure 4C). The atomic arrangement from a simulated HRTEM image with an overlapped projection of atoms taken from the [011] zone axis is in good agreement with the pattern observed experimentally (Figure 4D). The EDX mappings also confirmed the core–shell structure of the Ru_hcp_@Rh_fcc_ nanocrystals, with Ru atoms confined to the center and Rh atoms primarily distributed across the entire nanocrystal (Figure 4E). These results verified that the Rh shell obtained under the island growth mode due to the fast injection rate did not replicate the phase of Ru_hcp_ seeds but took its intrinsically stable fcc phase. Taken together, fine‐tuning the injection rate of precursor plays an essential role in determining the growth mode and thus the eventual phase of the deposited Rh overlayers through the interplay of bulk (crystal phase) and surface energies (smoothness of the shell).

Beyond the injection rate of the precursor, the phase transition as a function of growth mode can be activated by varying the amount of precursor added to the growth solution.^[^ ^11^, ^13^, ^33^ ^]^ This synthetic parameter directly controls the critical thickness within which the deposited shell will follow the crystal structure of the underlying seed. Previous studies have found that the Pd shell deposited on Ru_hcp_ seeds reverted to their native fcc phase at a threshold of ca. 5 atomic layers.^[^ ^13^ ^]^ To investigate this trend, we tuned the Rh shell thickness by varying the amount of Rh(III) precursor while maintaining a fixed overall injection volume of 10 mL and a constant concentration of Ru_hcp_ seeds. By changing the precursor amount from 9.6 mg (used in the standard protocol) to 6.4, 12.8, and 16 mg, the as‐obtained Ru@Rh nanocrystals featured a shell thickness of ca. 0.53, 1.11, and 1.27 nm (estimated based on the size of the Ru seeds and final core–shell product), corresponding to ca. 2–3, 4–5, and 5–6 atomic layers of Rh deposition (Figure S6, Supporting Information). The XRD patterns show that the Rh shell maintained a metastable hcp phase for thicknesses up to 1.11 nm (equivalence to ca. 4–5 atomic layers) before reverting to native fcc‐Rh at shell thickness of 1.27 nm and beyond (above 5 atomic layers) (Figure S6A–C, Supporting Information). This relationship between shell thickness and phase transition could be attributed to a shift from layer‐by‐layer to island growth mode, as evidenced by morphological changes observed in TEM images (Figure S6D–F, Supporting Information). With increased precursor amounts and thus accelerated reduction kinetics, the island growth was triggered since the atom deposition rate surpassed the surface diffusion rate, resulting in rough Rh shells. In this system, the high surface energy associated with abundant undercoordinated atoms on the surface, coupled with the high bulk energy of the metastable hcp‐Rh in the first few layers, forced subsequent Rh atoms to follow the thermodynamically favorable fcc stacking sequence to alleviate the in‐built strain. Therefore, an increase in Rh shell thickness beyond a threshold of ca. 5 atomic layers on Ru_hcp_ seeds induces an hcp‐to‐fcc phase transformation due to the shift in growth mode and balance between bulk and surface energies.

### 2.2 Evaluation of the Thermal Stability

**Figure 5:**
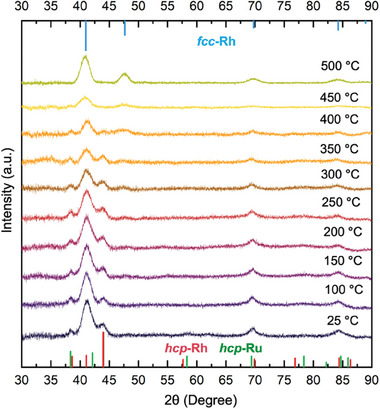
In situ XRD patterns of the Ru_hcp_@Rh_hcp_ nanocrystals measured under Ar atmosphere in the temperature range between 100 and 500 °C, indicating that the metastable hcp‐Rh structure could be preserved up to 400 °C.

Nanocrystals based on Rh are promising catalysts for a myriad of high‐temperature applications, including exhaust treatment and syngas production.^[^ ^42^, ^43^ ^]^ Therefore, understanding the thermal stability of the metastable hcp phase of Rh is crucial for evaluating their efficacy in these environments. We investigated the thermal behavior of the as‐obtained Ru_hcp_@Rh_hcp_ core–shell nanocrystals with a shell thickness of ca. 0.9 nm by heating them to various temperatures ranging from 25 to 450 °C under an Ar atmosphere. In situ XRD analysis was employed to monitor their phase evolution upon thermal activation (**Figure** **5**). The metastable hcp phase of the Rh shell was well‐preserved when heated to 350 °C, as evidenced by the distinct (101̅0), (0002), and (101̅1) peaks observed in the 38–45° range. As the temperature reached 400 °C, the XRD pattern continued to be dominated by hcp peaks, albeit with reduced intensities. Concurrently, a weak (200) peak indicative of fcc‐Rh emerged at 47.8°, suggesting the coexistence of hcp and fcc phases within the Rh shell. Upon heating to 450 °C, the hcp‐to‐fcc phase transformation became pronounced, with the characteristic hcp‐Rh peaks significantly diminished and those of fcc‐Rh prevailing in the XRD pattern. Further heating to 500 °C led to increased intensities of all fcc‐Rh peaks, which could be attributed to an increased percentage of fcc phase, partial recrystallization, and/or surface rearrangement of the nanocrystals.^[^ ^44^, ^45^ ^]^ This transition marks a complete reversion of the Rh shell to its native fcc phase, consistent with the equilibrium state of bulk Rh. Collectively, these in situ XRD data demonstrate that Ru_hcp_@Rh_hcp_ nanocrystals retained their metastable hcp‐Rh phase up to 400 °C, suggesting their potential functionality in high‐temperature catalytic processes.

### 2.3 Evaluation of the Catalytic Performance

With similar size and composition but different packing sequences, the as‐obtained Ru@Rh nanocrystals featuring polymorphism can serve as model systems to investigate the impact of the crystal phase on the electrocatalytic performance. As a proof‐of‐concept application, we evaluated the Ru@Rh nanocrystals made of fcc‐ or hcp‐Rh in the shell toward EOR since Rh has been proven as an effective catalytic material for this anodic reaction in direct ethanol fuel cells.^[^ ^36^, ^46^, ^47^, ^48^ ^]^ A dual‐pathway mechanism has been proposed for EOR on Rh‐based surfaces in alkaline media: the C_1_ pathway, where ethanol is fully oxidized to CO_2_ or carbonate through the cleavage of the C─C bond, transferring a total of 12 electrons; and the C_2_ pathway, where ethanol undergoes incomplete oxidation to acetic acid or acetate, transferring only 4 electrons.^[^ ^49^ ^]^ The different atomic arrangements on hcp‐ and fcc‐Rh shells could potentially impact their catalytic activity and selectivity toward these pathways in EOR by modulating the electronic structure of the catalyst's surface and the binding strength of the reaction intermediates.^[^ ^13^, ^16^, ^25^ ^]^

**Figure 6:**
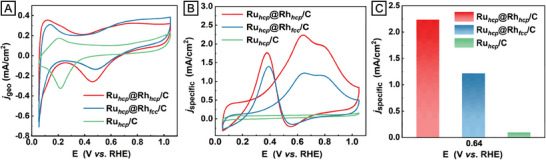
Electrocatalytic performance of the Ru@Rh nanocrystals with different crystal phases and Ru_hcp_ nanocrystal seeds toward EOR. A) CV curves and B) EOR specific activities of the three catalysts. C) Comparison of the specific activities of the three catalysts at a potential of 0.64 V versus RHE in the forward scans.

The EOR catalytic performance of the as‐synthesized Ru_hcp_@Rh_hcp_ and Ru_hcp_@Rh_fcc_ nanocrystals, with a similar shell thickness of ca. 0.9 nm, was compared against monometallic Ru_hcp_ nanocrystals of ca. 6.7 nm serving as seeds in the standard protocol. Figure S7 (Supporting Information) shows the Cu underpotential deposition (Cu_UPD_) stripping curves of the three catalysts recorded in an aqueous electrolyte containing 0.5 m H_2_SO_4_ and 5 × 10^−3^ m CuSO_4_. The electrochemical surface areas (ECSAs) of Ru_hcp_@Rh_hcp_, Ru_hcp_@Rh_fcc_, and Ru_hcp_ nanocrystals were derived by integrating the stripping charges of Cu_UPD_ and summarized in Table S1 (Supporting Information). The distinct surface compositions among the three catalysts were also revealed by analyzing features of cyclic voltammetry (CV) curves recorded in 0.1 m HClO_4_ (**Figure** **6**A). Specifically, the CV curve of Ru_hcp_ nanocrystals displayed a cathodic peak at ca. 0.24 V (vs reversible hydrogen electrode, RHE), corresponding to the reduction of Ru─O.^[^ ^50^ ^]^ For Ru_hcp_@Rh_hcp_ and Ru_hcp_@Rh_fcc_ nanocrystals, the cathodic peak for reduction of Rh─O was prominent in the range of 0.45–0.65 V, consistent with voltammetric features of Rh‐based catalysts observed in previous reports.^[^ ^51^ ^]^ The absence of a Ru─O reduction peak centered at 0.25–0.35 V implied that the surface of these Ru@Rh core–shell nanocrystals was predominantly composed of Rh, rather than a Ru─Rh alloy. This complete encapsulation by Rh not only prevents the Ru cores from oxidizing but also ensures that the catalytic processes are primarily mediated by the Rh surface.

Figure 6B shows the EOR polarization curves of the three catalysts recorded in an aqueous electrolyte containing 1 m KOH and 1 m ethanol. Utilizing Ru_hcp_ nanocrystals as the catalyst, no oxidation peaks for ethanol were observed in either the forward or backward scans, suggesting the relative inertness of Ru in catalyzing EOR. As such, we hypothesized that the catalytic activity towards EOR of Ru_hcp_@Rh_hcp_ and Ru_hcp_@Rh_fcc_ nanocrystals could be mainly attributed to the Rh shell with different crystal phases. In the forward scans of Ru_hcp_@Rh_hcp_ and Ru_hcp_@Rh_fcc_ nanocrystals, two characteristic oxidation peaks could be observed at ca. 0.64 and 0.8 V versus RHE. According to previous reports, the anodic peak at 0.64 V could be attributed to the oxidation of ethanol to acetaldehyde and CO_2_, while the anodic peak at 0.8 V corresponds to the involvement of acetic acid formation.^[^ ^36^, ^39^, ^49^ ^]^ The ECSA‐normalized peak current density in the forward scans (*j*_f_) is used to estimate the specific activity of catalysts (*j*_specific_). The Ru_hcp_@Rh_hcp_ nanocrystals achieved the highest *j*_specific_ value among the tested catalysts over the whole potential range. Their EOR specific activities and mass activities are summarized in Table S1 (Supporting Information). The *j*_specific_ values at 0.64 V versus RHE of three catalysts were used to quantitatively compare their EOR activity. The Ru_hcp_@Rh_hcp_ exhibited the highest EOR specific activity (2.2 mA cm^−2^), which was 1.8 and 22 times higher than those of the Ru_hcp_@Rh_fcc_ (1.2 mA cm^−2^) and Ru_hcp_ (0.1 mA cm^−2^) counterparts, respectively (Figure 6C). This result validated that the metastable hcp‐Rh phase on Ru@Rh core–shell nanocrystals was more active than the fcc‐Rh phase in catalyzing EOR.

The adsorbed ethanol‐derived CO is a notorious intermediate blamed for poisoning Rh active sites during EOR. The ECSA‐normalized peak current density for the backward scans (*j*_b_) is associated with further oxidation of the freshly‐formed carbonaceous intermediates (e.g., CO) built up on the catalyst's surface from the forward scans.^[^ ^49^ ^]^ The ratio of *j*_f_ to *j*_b_ (*j*_f_/*j*_b_) has been widely utilized as an indicator for the tolerance of the catalyst to CO poisoning.^[^ ^49^, ^52^ ^]^ The *j*_f_/*j*_b_ ratio of Ru_hcp_@Rh_hcp_ nanocrystals was found to be higher than that of Ru_hcp_@Rh_fcc_ nanocrystals (1.26 vs 0.87), suggesting their efficient oxidation or removal of CO_ad_ and/or CH*_x_* intermediates (Figure 6B and Table S1, Supporting Information). We also characterized Ru_hcp_@Rh_hcp_ nanocrystals after electrochemical measurements to assess their stability in terms of crystal phase and morphology. Both the pseudo‐spherical shape and metastable hcp‐Rh phase of the Ru_hcp_@Rh_hcp_ nanocrystals were retained as confirmed by HRTEM and FFT analyses (Figure S8, Supporting Information). Collectively, the enhanced activity, high CO tolerance, and excellent structural stability endow Ru_hcp_@Rh_hcp_ nanocrystals with promising characteristics as catalysts for EOR.

With similar size, composition, and shell thickness, the enhanced catalytic performance of Ru_hcp_@Rh_hcp_ nanocrystals when benchmarked against Ru_hcp_@Rh_fcc_ counterparts could be attributed to the formation of metastable hcp‐Rh phases which can influence the binding strength of reaction intermediates, as suggested by previous studies. In contrast to fcc nanocrystals mainly enclosed by the {111} facets, hcp nanocrystals typically possess a large percentage of open, less closed‐packed facets (e.g., {101̅0} and {101̅0}), which accounted for more than 60% of the total surface area.^[^ ^53^ ^]^ Previous DFT calculations have demonstrated that such open Rh surfaces have stronger binding strength, lower dehydrogenation barrier, and lower reaction barrier of C─C scission of ethanol compared to the close‐packed facets.^[^ ^46^, ^54^ ^]^ As such, we hypothesized that the enhanced performance of Ru_hcp_@Rh_hcp_ nanocrystals towards EOR can potentially be attributed to the exposure of these abundant open facets with favorable binding to intermediate species and thus facilitating the C─C bond breaking for the complete oxidation of ethanol to CO_2_. However, it should be noted that the surfaces of Ru_hcp_@Rh_hcp_ and Ru_hcp_@Rhfcc nanocrystals are covered by various types of facets due to their morphologies. This surface inhomogeneity complicates the attribution of specific catalytic properties to individual facets. Therefore, the precise surface structures and the exact mechanisms underlying the enhanced activity on Rh‐based nanocrystals with metastable hcp phase require more in‐depth analysis. Further characterizations using in situ electrochemical spectroscopy and computational studies will be essential to fully understand the oxidation mechanism of ethanol on different surfaces of the hcp‐Rh structure.

## 3 Conclusion

In summary, we have successfully synthesized Ru@Rh core–shell nanocrystals with a uniform Rh shell in the metastable hcp phase. The use of Ru_hcp_ seeds was crucial for activating the templating effect necessary for the epitaxial growth of Rh overlayers, facilitated by the small lattice mismatch between Ru and Rh, the optimal nanocrystal size for surface diffusion of Rh adatoms, and the structural stability during synthesis. By employing a slow dropwise titration of the Rh(III) precursor at an elevated temperature of 160 °C, a layer‐by‐layer growth mode was achieved, resulting in the formation of a uniform and conformal hcp‐Rh shell. This outcome was attributed to the balance between low surface energy from smooth overlayers and high bulk energy of the metastable phase. On the basis of phase dependence on growth mode, switching to a faster injection rate of the precursor led to the formation of a rough Rh shell in the conventional fcc phase, driven by the need to offset the gain in surface energy with a decrease in bulk energy. The metastable hcp‐Rh structure of Ru_hcp_@Rh_hcp_ nanocrystals could be preserved up to 400 °C, as established by in situ XRD analyses. When used as catalysts, the Ru_hcp_@Rh_hcp_ nanocrystals exhibited enhanced activity towards EOR compared to their Ru_hcp_@Rh_fcc_ and Ru_hcp_ counterparts. This work presents a robust and facile route for fabricating Rh‐based nanocrystals featuring polymorphism, opening new possibilities for exploring the phase–property relationship in various catalytic processes. The seed‐mediated growth method, integrating both thermodynamic and kinetic control, could be extended to other metal systems, thereby expanding the library of crystal phases in noble‐metal nanocrystals for diverse applications.

## 4 Experimental Section

### Chemicals and Materials

Ruthenium(III) acetylacetonate (Ru(acac)_3_, 97%), rhodium(III) acetylacetonate (Rh(acac)_3_, 97%), and poly(vinylpyrrolidone) (PVP, with an average molecular weight of 55k), acetic acid (99.7%), potassium hydroxide (KOH, 85%), sulfuric acid (H_2_SO_4_, 99.999%), perchloric acid (HClO_4_, 70%), Cu(II) sulfate (CuSO_4_, anhydrous powder, 99.99%), and Nafion (5 wt%) were purchased from Sigma‐Aldrich. Ethylene glycol (EG, 99%) was obtained from J. T. Baker. Ethanol (99.5%) and acetone (99.5%) were purchased from VWR. Carbon black was purchased from Cabot (VULCANXC72R). All chemicals were used as received. Aqueous solutions were prepared using deionized water with a resistivity of 18.2 MΩ cm at room temperature.

### Characterizations

Transmission electron microscopy (TEM) images were taken on a Hitachi HT7700 microscope operated at 120 kV. The sample for TEM analysis was prepared by drop‐casting an ethanol suspension of the nanocrystals on a carbon‐coated copper grid, followed by drying under ambient conditions. High‐resolution transmission electron microscopy (HRTEM) images were obtained using an FEI Tecnai G2 F30 TEM operated at 300 kV. Scanning transmission electron microscopy (STEM) images were acquired using a Cs‐corrected Hitachi HD2700 STEM operated at 200 kV. Energy‐dispersive X‐ray spectroscopy (EDX) data were acquired using a Cs‐corrected FEI Titan 80/300 kV TEM/STEM at Oak Ridge National Laboratory (ORNL). X‐ray diffraction (XRD) patterns were recorded on a PANalytical X'Pert PRO Alpha‐1 diffractometer using a 1.8 kW ceramic copper tube source. In situ XRD measurements were carried out on Rigaku SmartLab XE equipped with an Anton Paar TTK600 stage for precise temperature control. The X‐ray photoelectron spectroscopy (XPS) data were collected on a Thermo K‐Alpha spectrometer with an Al Kα source. An inductively coupled plasma mass spectrometer (ICP‐MS, Perkin Elmer, NexION 300Q) was used to determine the metal content in the sample.

### Synthesis of Ru_hcp_ Nanocrystal Seeds

The procedure for synthesizing Ru_hcp_ nanocrystals was adapted from a previously published protocol with minor modifications.^[^ ^13^, ^18^ ^]^ In a typical synthesis, 7.5 mg of Ru(acac)_3_ and 50 mg of PVP were mixed in 5 mL of EG hosted in a 20 mL glass vial. The vial was then heated in an oil bath held at 180 °C under magnetic stirring at 400 rpm. After 2 h, the reaction was quenched by immersing the vial in an ice‐water bath. Simultaneously, a separate precursor mixture was prepared by dissolving 300 mg of Ru(acac)_3_ and 100 mg of PVP in 10 mL of EG. This mixture was preheated to 95 °C under magnetic stirring at 400 rpm until the Ru(III) precursor completely dissolved, yielding a reddish solution. Following the quenching step, the vial containing the initial 3 nm Ru_hcp_ nanocrystals was reheated at 180 °C for 10 min to reinitiate the reaction conditions. Afterwards, 10 mL of the newly prepared precursor solution in EG was injected into this vial at a rate of 4.0 mL h^−1^ using a syringe pump. Once all the precursor solution had been added, the reaction mixture continued to be held at 180 °C for another 2h and then quenched with an ice‐water bath. The resulting suspension of Ru_hcp_ nanocrystals in EG (ca. 4.93 mg mL^−1^) was used directly as seeds for the subsequent growth of the Rh shell, without further purification or treatment.

### Synthesis of Ru_hcp_@Rh_hcp_ and Ru_hcp_@Rh_fcc_ Nanocrystals

In a typical synthesis, 0.5 mL of the as‐synthesized Ru_hcp_ nanocrystal seeds in EG was mixed with 0.5 mL of EG and 50 mg of PVP hosted in a 20 mL glass vial. The mixture was then heated at 160 °C for 15 min under magnetic stirring of 400 rpm to ensure uniform heating and dispersion. Concurrently, 9.6 mg of Rh(acac)_3_ and 10 mg of PVP were dissolved in 10 mL of EG by heating at 95 °C for 15 min under magnetic stirring of 400 rpm, until a homogenous yellow solution is obtained. The Rh(III) precursor solution was then injected into the heated mixture of Ru_hcp_ nanocrystal seeds at a rate of 0.5 mL h^−1^ using a syringe pump. After the complete addition of the precursor solution, the reaction was allowed to proceed for another 2 h before quenching in an ice‐water bath. The solid products were collected by precipitation with acetone (acetone/EG = 3:1 v/v) and washed three times with a mixture of acetone and ethanol (3:1 v/v) under centrifugation. The synthesis of Ru_hcp_@Rh_fcc_ nanocrystals followed a similar protocol to that of the Ru_hcp_@Rh_hcp_ nanocrystals, except that the Rh(acac)_3_ precursor solution in EG was injected at an increased rate of 4 mL h^−1^.

### Thermal Stability of the hcp‐Rh Structure

The thermal stability of the hcp‐Rh structure in the Ru_hcp_@Rh_hcp_ core–shell nanocrystals was evaluated using in situ XRD performed on Rigaku SmartLab XE. The sample was gradually heated from 25 to 500 °C at a rate of 5 °C s^−1^, employing an Anton Paar TTK600 heating stage. At each designated temperature within this range, the XRD pattern was recorded for 1 h, spanning 2*θ* angles from 30° to 90°. All the measurements were carried out under an Ar atmosphere to prevent oxidation of the sample.

### Preparation of the Catalysts

In a standard process, carbon black was dispersed in ethanol at a concentration of 1 mg mL^−1^ and sonicated in an ice bath for 1 h. Subsequently, an ethanol suspension of the synthesized nanocrystals is added to achieve ca. 20% total metal loading by weight relative to the carbon black. The mixture was further sonicated in an ice bath for another 1 h and then centrifuged at 12 000 rpm for 10 min to collect the catalyst powder. To clean the surface of the particles, the catalysts were then dispersed in 5 mL of acetic acid, heated at 60 °C for 3 h, and washed three times with ethanol. After that, the catalyst was dried in an oven held at 80 °C for 5 h. The final catalyst ink was prepared by redispersing 2.5 mg of the dried carbon‐supported nanocrystals in 2.5 mL of ethanol containing 10 µL of Nafion, followed by sonication in an ice bath for 1 h.

### Electrochemical Measurements

The electrochemical measurements were conducted in a three‐electrode cell connected to a CHI 600E electrochemical workstation at room temperature. For all measurements, a glassy carbon (GC, 5 mm in diameter) and Pt mesh (1 × 1 cm^2^) served as working and counter electrodes, respectively. A reversible hydrogen electrode (RHE, HydroFlex, Gasketel) was used as a reference electrode for the cyclic voltammetry (CV) scanning in aqueous electrolytes containing HClO_4_ or KOH. For Cu underpotential deposition (UPD), a saturated calomel electrode (SCE) was employed as a reference electrode. The GC electrode was prepared by polishing with 0.3‐ and then 0.05 µm Al_2_O_3_ powders, followed by rinsing with water and ethanol. Afterward, 10 µL of the catalyst ink was dropped on the GC electrode and dried at room temperature. Unless otherwise noted, all potentials were presented with reference to RHE, and all data were corrected with an 85% iR compensation to account for the voltage drop between the working electrode and reference electrode.

Prior to electrochemical testing, all solutions were purged and saturated with Ar. The catalyst was first cycled in an Ar‐saturated HClO_4_ solution (0.1 m) between 0.05 and 1.05 V_RHE_ at 500 mV s^−1^ for several hundred cycles to clean the surface until a stable CV curve was achieved. CV curves were subsequently recorded at a scan rate of 50 mV s^−1^ in the same aqueous electrolyte solution. The electrode was then cycled in an Ar‐saturated solution containing 1 m KOH between 0.05 and 1.05 V_RHE_ at a scan rate of 500 mV s^−1^ until the current density reached a stable value. Afterward, the ethanol oxidation activity test was assessed in a mixture of 1 m ethanol and 1 m KOH solution between 0.05 and 1.05 V_RHE_ at a scan rate of 50 mV s^−1^.

The electrochemical active surface areas (ECSAs) of the three catalysts were further determined using the Cu_UPD_ method.^[^ ^13^ ^]^ Briefly, the electrode was first cycled in Ar‐saturated 0.5 m H_2_SO_4_ from 0.02 to 1.02 V_RHE_ at a scan rate of 10 mV s^−1^ after cleaning the electrode at 500 mV s^−1^ for several hundred cycles. Then, the potential was fixed at 0.26 V_RHE_ for 100 s in an Ar‐saturated aqueous electrolyte containing 0.5 m H_2_SO_4_ and 5 × 10^−3^ m CuSO_4_, followed by a linear scan from 0.26 to 1.02 V_RHE_ to collect the Cu_UPD_ curve. The ECSAs were calculated by integrating the stripping charge of Cu_UPD_, subtracting the charge obtained under the same conditions in 0.5 m H_2_SO_4_, and assuming a charge density of 420 µC cm^−2^ for all catalysts.

## Conflict of Interest

The authors declare no conflict of interest.

## Supporting information



Supporting Information

## Data Availability

The data that support the findings of this study are available from the corresponding author upon reasonable request.
