# Supplementary material for: Facile Synthesis of Rhodium‐Based Nanocrystals in a Metastable Phase and Evaluation of Their Thermal and Catalytic Properties
Source: Small Methods. 2024 Oct 22;9(3):2401143. doi: 10.1002/smtd.202401143 (PMC11926511; doi:10.1002/smtd.202401143)
Supplement: Supplementary file 1 — Supporting Information [file SMTD-9-2401143-s001.docx]

Supporting Information

**Facile Synthesis of Rhodium-Based Nanocrystals in a Metastable Phase and Evaluation of Their Thermal and Catalytic Properties**

Quynh N. Nguyen,^†^ Kei Kwan Li,^†^ Yong Ding,^‡^ Annemieke Janssen,^†^ Zhennan Huang,^§^ Miaofang Chi,^§^ and Younan Xia^†,¶,^*

^†^School of Chemistry and Biochemistry, Georgia Institute of Technology, Atlanta, Georgia 30332, United States

^‡^School of Materials Science and Engineering, Georgia Institute of Technology, Atlanta, Georgia 30332, United States

^§^Center for Nanophase Materials Science, Oak Ridge National Laboratory, Oak Ridge, Tennessee 37831, United States

^¶^The Wallace H. Coulter Department of Biomedical Engineering, Georgia Institute of Technology and Emory University, Atlanta, Georgia 30332, United States

^*^Address correspondence to [younan.xia@bme.gatech.edu](mailto:younan.xia@bme.gatech.edu)

**
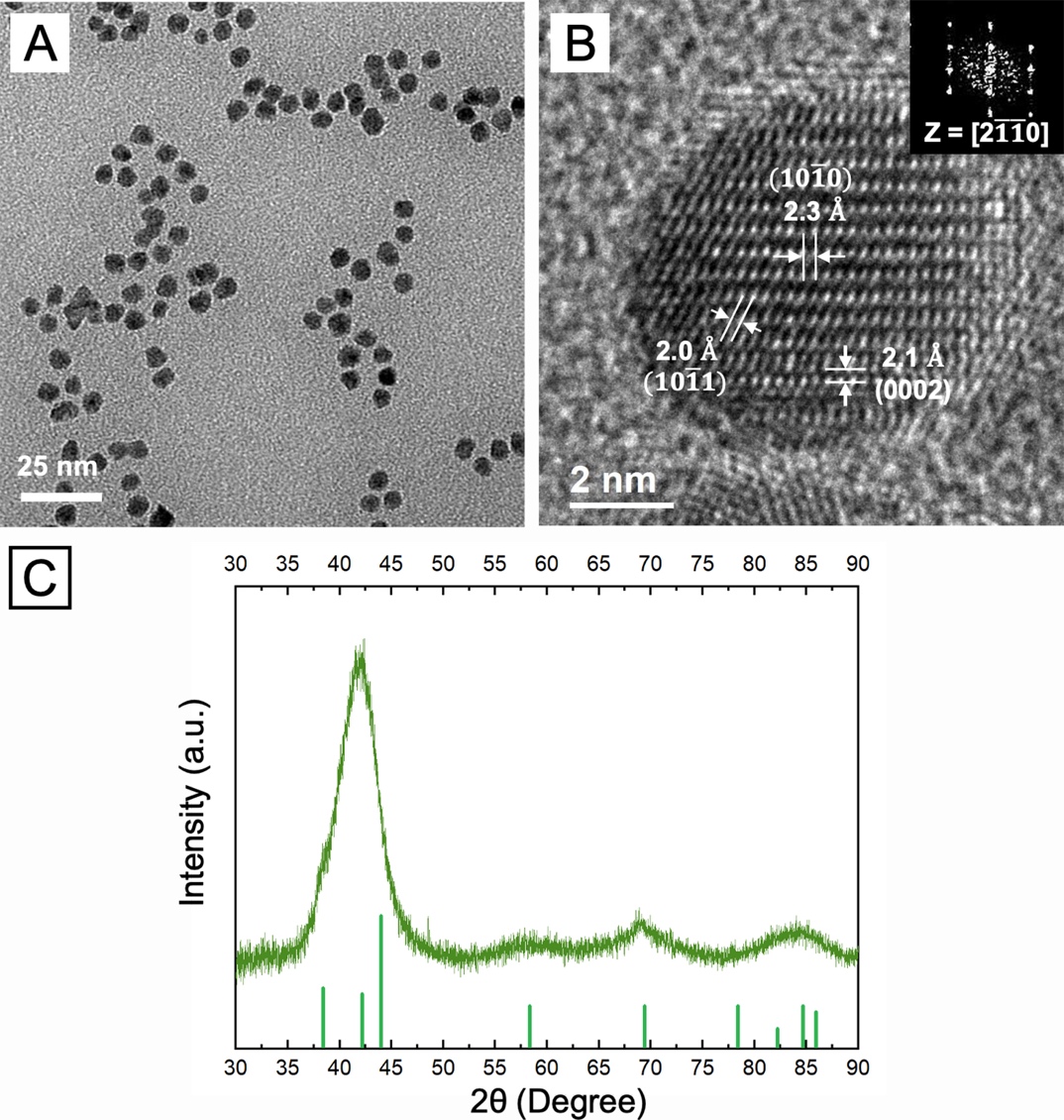
**

**Figure S1.** Structural characterizations of the Ru*_hcp_* nanocrystals serving as seeds for the deposition of Rh. (A) TEM image, (B) HRTEM image with the inset as the corresponding FFT pattern, and (B) XRD pattern of the Ru nanocrystals, confirming their *hcp* crystal structure. The green lines incorrespond to the characteristic peaks of *hcp*-Ru (JCPDS No. 06-0663).


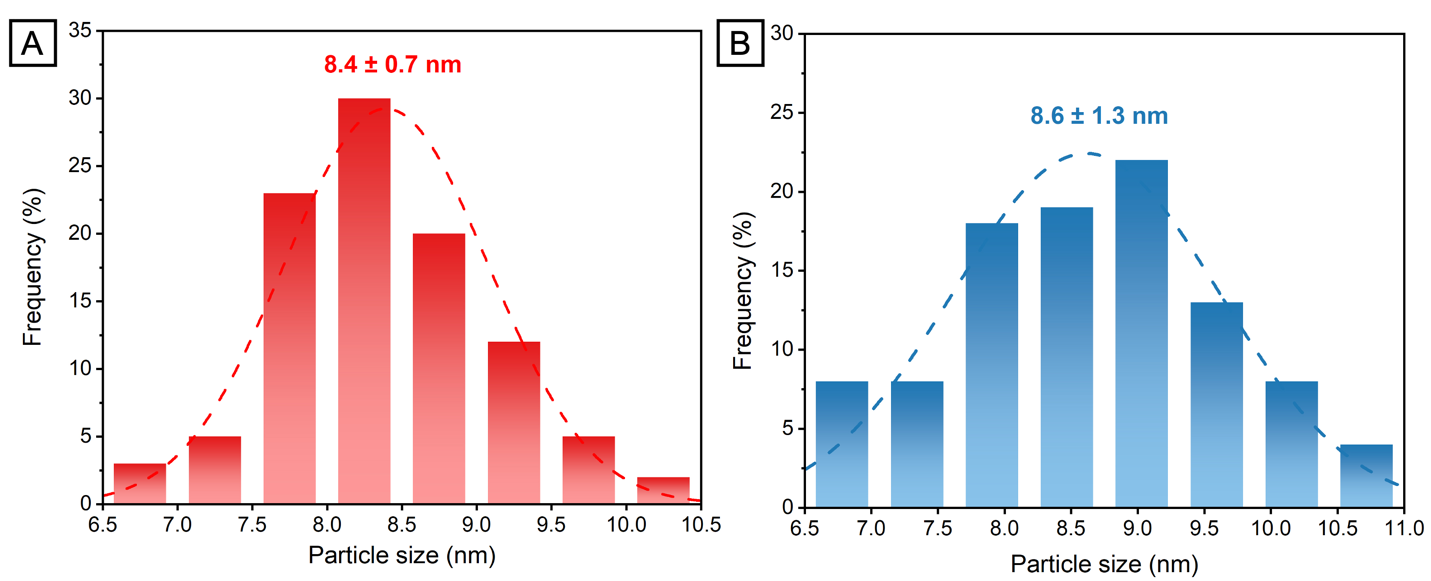


**Figure S2.** Size distributions of (A) Ru*_hcp_*@Rh*_hcp_* and (B) Ru*_hcp_*@Rh*_fcc_* nanocrystals.


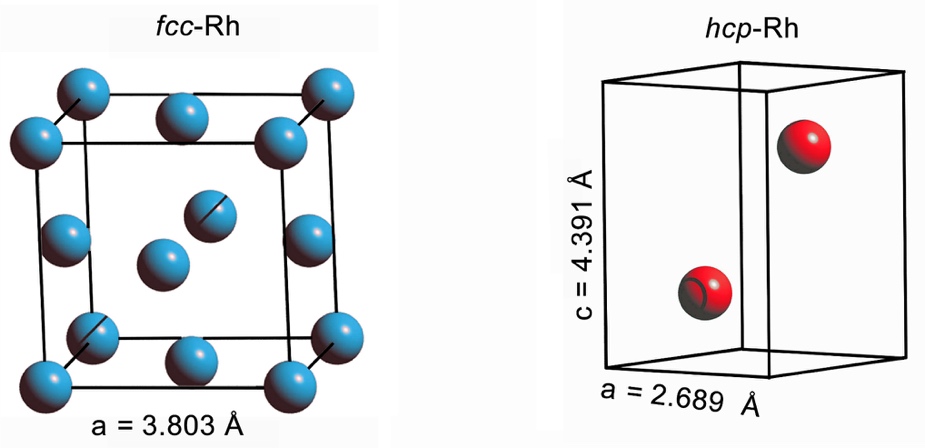


**Figure S3.** Schematic diagrams of the unit cells of *fcc*- and *hcp*-Rh structures. The lattice parameters of an *hcp*-Rh structure (a = 2.689 Å, c = 4.391 Å) were calculated based on those of *fcc*-Rh structure (a = 3.803 Å, JCPDS No. 05-0685) using the relationship a*_fcc_* = $\sqrt{2}$ a*_hcp_* and c*_hcp_* = 2$\sqrt{6}$/3 a*_hcp_*.


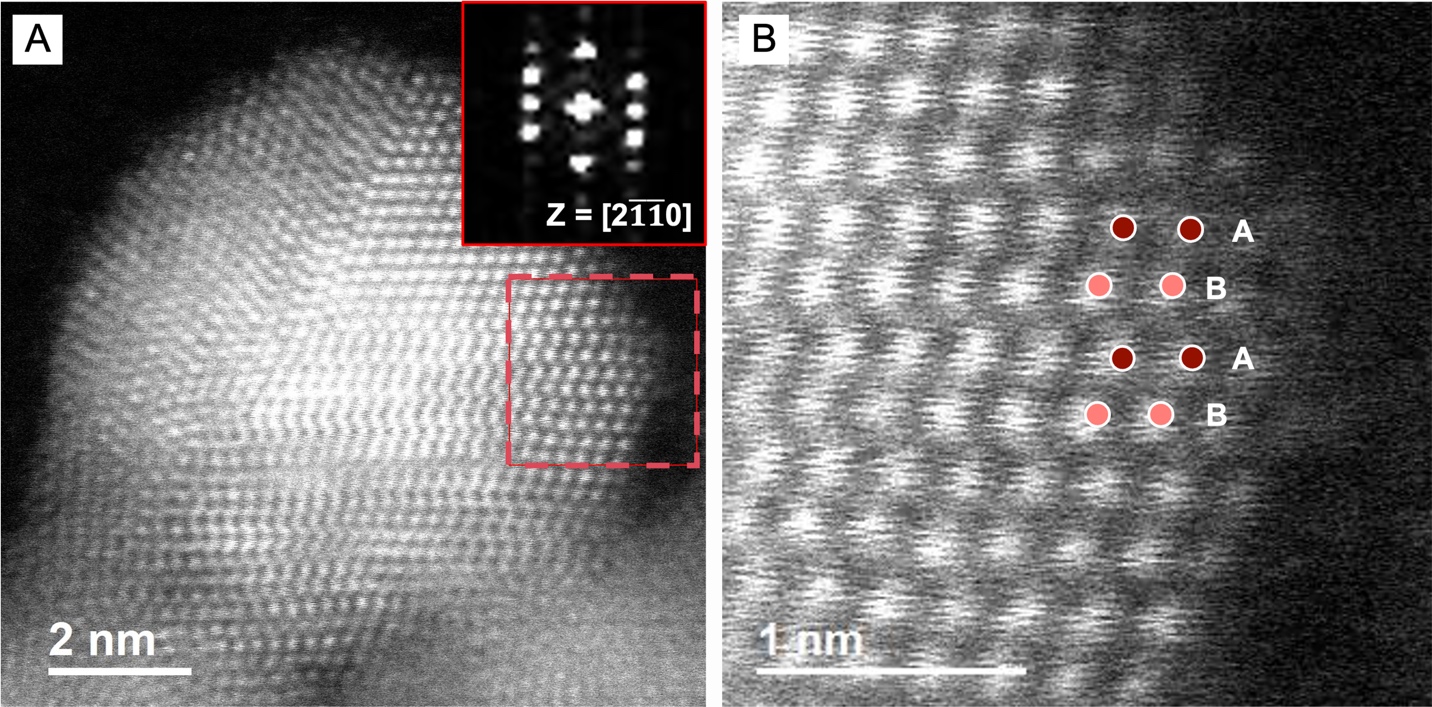


**Figure S4.** (A) HAADF-STEM image and (B) atomic resolution HAADF-STEM image of an individual Ru*_hcp_*@Rh*_hcp_* core-shell nanocrystal. The inset in (A) shows the corresponding FFT pattern of the red boxed region.


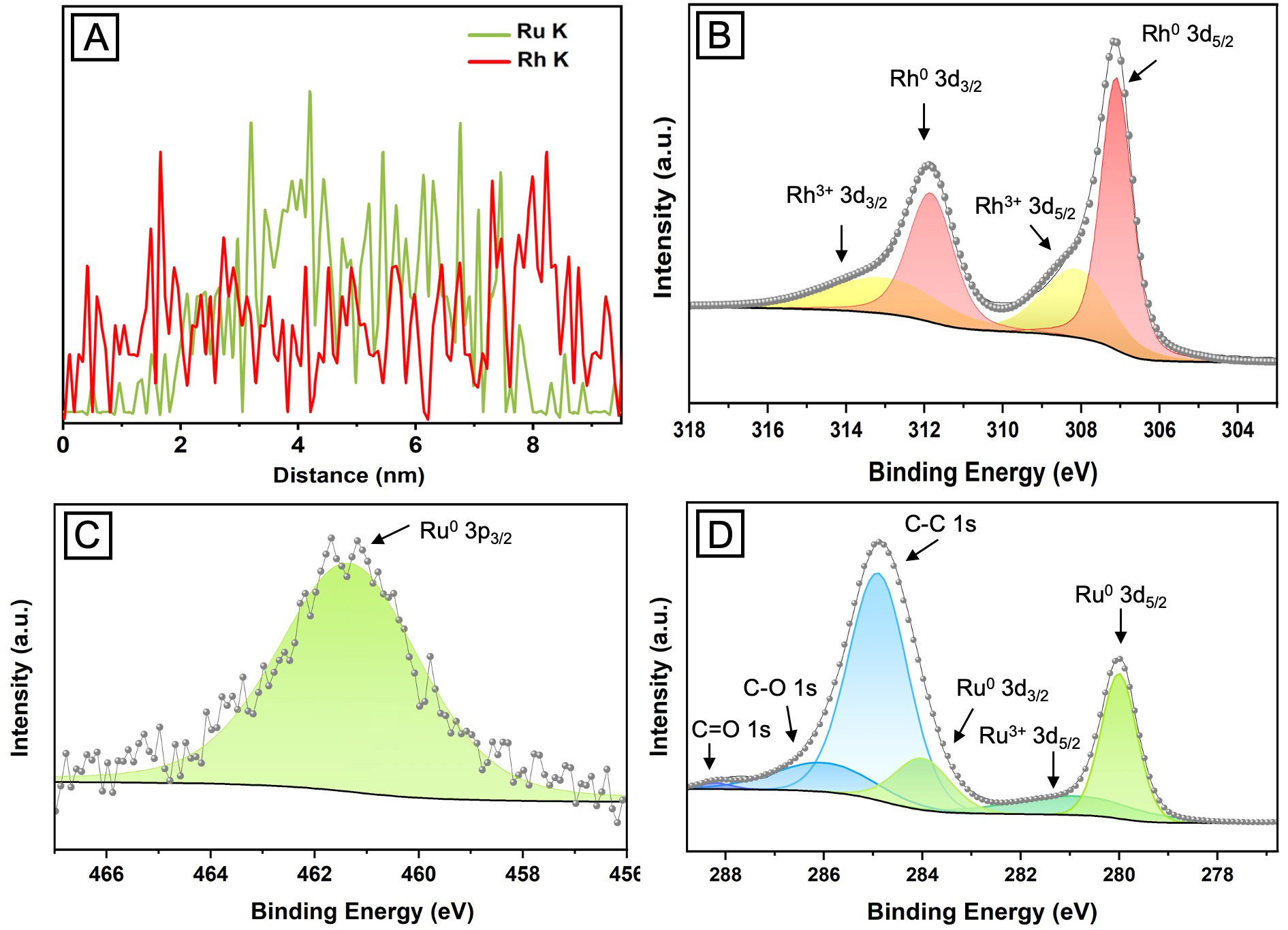


**Figure S5.** (A) Line profiles of EDX intensities across the Ru*_hcp_*@Rh*_hcp_* core-shell nanocrystals as shown in Figure 3E. (B) Rh 3d, (C) Ru 3p, and (D) Ru 3d XPS spectra of the Ru*_hcp_*@Rh*_hcp_* core-shell nanocrystals. The zero-valent component is in dominance for each metal. Note that the sample for XPS analysis was deposited on a glass slide and the C 1s signals may originate from the surfactants adsorbed on the surface of the nanocrystals.

**
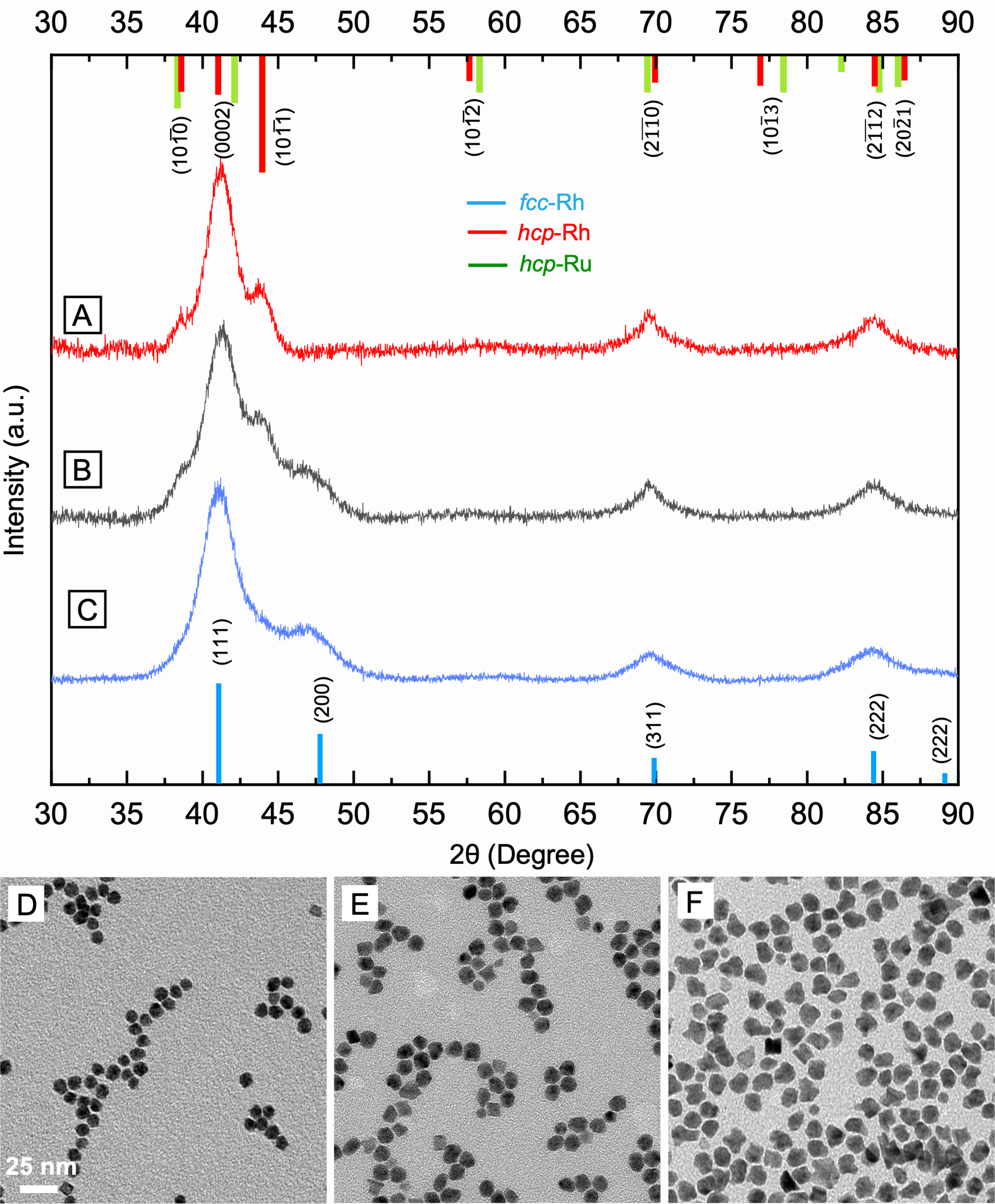
**

**Figure S6.** (A–C) XRD patterns and (D–F) TEM images recorded from the Ru@Rh nanocrystals with different shell thicknesses: (A, D) 0.53, (B, E) 1.11, and (C, F) 1.27 nm. The red, blue, and green lines correspond to the characteristic peaks of *hcp*-Rh (simulated), *fcc*-Rh (JCPDS No. 05-0685), and *hcp*-Ru (JCPDS No. 06-0663), respectively.


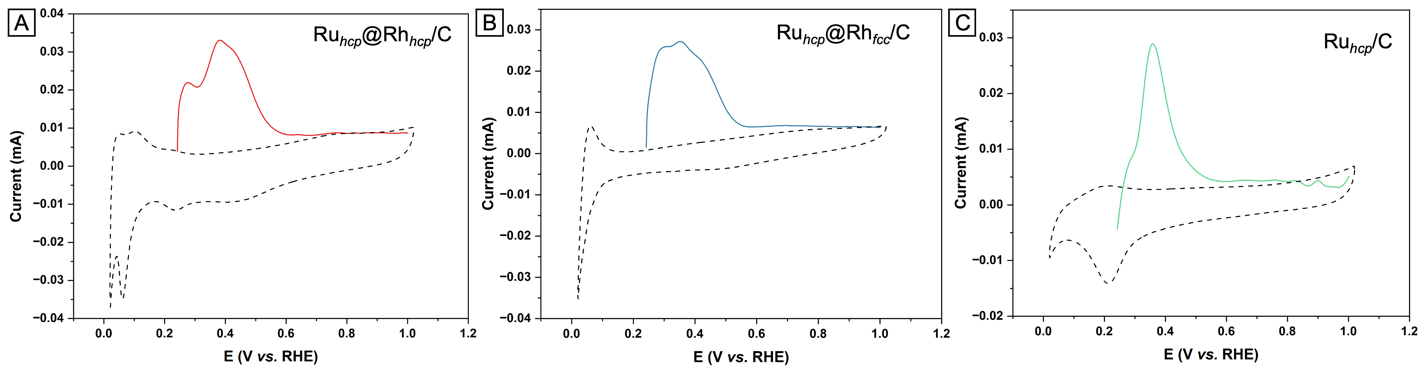


**Figure S7.** Cu underpotential deposition (Cu_UPD_) curves (solid lines, recorded in 0.5 M H_2_SO_4_ and 5 mM CuSO_4_ at 10 mV s^−1^) and cyclic voltammetry curves (dashed lines, recorded in 0.5 M H_2_SO_4_ at 10 mV s^−1^) of (A) Ru*_hcp_*@Rh*_hcp_*, (B) Ru*_hcp_*@Rh*_fcc_*, and (C) Ru*_hcp_* nanocrystals.

**Table** **S1.** Summary of the ECSAs derived from the stripping changes of underpotentially deposited Cu, *j_specific_* or specific activities (SA), and mass activities (MA) of the three catalysts towards EOR shown in Figure S6 and Figure 6, respectively.

| Catalysts | Ru*_hcp_*@Rh*_hcp_* | Ru*_hcp_*@Rh*_fcc_* | Ru*_hcp_* |
| --- | --- | --- | --- |
| ECSA (m^2^/g) | 28.7 | 32.2 | 50.4 |
| SA at 0.64 V (mA cm^–2^) | 2.22 | 1.23 | 0.10 |
| MA at 0.64 V (A mg^–1^) | 0.64 | 0.40 | 0.05 |
| Anodic peak potential (V) | 0.64 *^a^* \| 0.79 *^b^* | 0.66 *^a^* \| 0.80 *^b^* | – |
| *j_f_* (mA cm^–2^) | 2.22 *^a^* \| 1.94 *^b^* | 1.22 *^a^* \| 1.14 *^b^* | – |
| Cathodic peak potential (V) | 0.38 | 0.39 | – |
| *j_b_* (mA cm^–2^) | 1.78 | 1.40 | – |
| *j_f_*/*j_b_* | 1.36 *^a^* \| 1.09 *^b^* | 0.87 *^a^* \| 0.81 *^b^* | – |

^a^ Obtained from the first anodic peak at *ca.* 0.64 V in Figure 6A.

^b^ Obtained from the second anodic peak at *ca.* 0.8 V in Figure 6A.


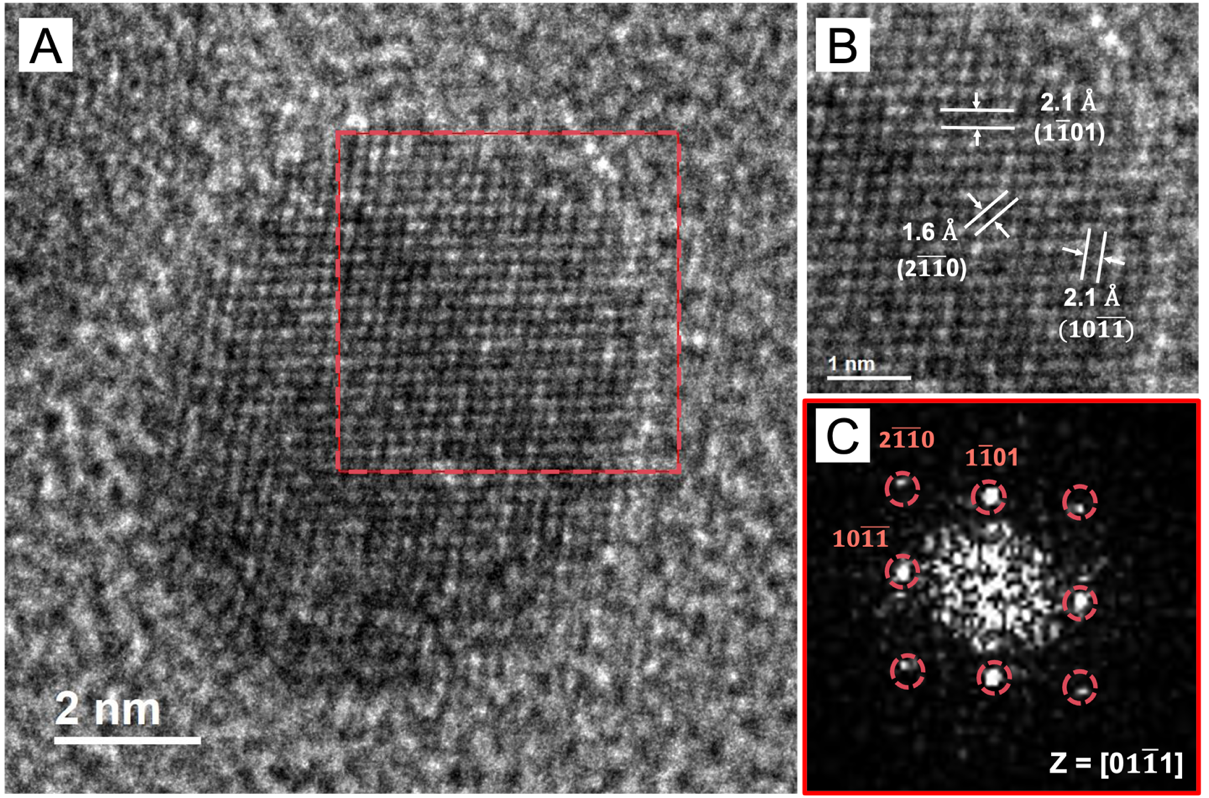


**Figure S8.** Characterizations of an individual Ru*_hcp_*@Rh*_hcp_* core-shell nanocrystal after EOR test: (A) HRTEM image, (B) atomic resolution HRTEM image, and (C) the corresponding FFT pattern of the red boxed region in (A) viewed along the [$01\bar{1}1]$ direction.
